# Potential Functional Food Products and Molecular Mechanisms of *Portulaca Oleracea* L. on Anticancer Activity: A Review

**DOI:** 10.1155/2022/7235412

**Published:** 2022-09-20

**Authors:** Pâmela Gomes de Souza, Amauri Rosenthal, Ellen Mayra Menezes Ayres, Anderson Junger Teodoro

**Affiliations:** ^1^Graduate Program in Food and Nutrition, Federal University of the State of Rio de Janeiro (UNIRIO), Av. Pasteur, 296, Urca, Rio de Janeiro, RJ, Brazil; ^2^Laboratory of Sensory and Consumer Science, Graduate Program in Food and Nutrition, Federal University of the State of Rio de Janeiro (UNIRIO), Av. Pasteur, 296, Urca, Rio de Janeiro, RJ, Brazil; ^3^Embrapa Food Technology, Av. das América, 29501, Guaratiba, Rio de Janeiro, RJ, Brazil; ^4^Departament of Nutrition and Dietetics, Universidade Federal Fluminense Rua Mário Santos Braga, 30 Niterói RJ, Brazil

## Abstract

*Portulaca oleracea* Linn. (*P. oleracea* L.) has recently gained attention as a functional food due to the chemical composition of this plant regarding bioactive compounds. The special attention to the use of *P. oleracea* as an ingredient in functional food products is also due to the promotion of sustainable food. It is an unconventional food plant, and its consumption may contribute to preserving biodiversity due to its cultivation in a polyculture system. Food sovereignty may be achieved, among other strategies, with the consumption of unconventional food plants that are more resistant in nature and easily cultivated in small places. *P. oleracea* grows spontaneously and may be found in streets and sidewalks, or it may be cultivated with seeds and cuttings propagation. The culinary versatility of *P. oleracea* opens up opportunities to explore the development of sustainable, functional food products. This mini-review shows that functional food products developed from *P. oleracea* are already available at the research level, but it is expected that more scientific literature focusing on the development of *P. oleracea* functional products with proven anticancer activities may be released in the near future. Polysaccharides, some phenolic compounds, alkaloids, and cerebrosides are associated with the inhibition and prevention of carcinogenesis through *in vitro* and *in vivo* investigations. The anticancer activities of *P. oleracea*, its bioactive compounds, and the involved molecular mechanisms have been reported in the literature. The importance of further elucidating the cancer inhibition mechanisms is in the interest of forthcoming applications in the development of food products with anticancer properties for implementation in the human diet.

## 1. Introduction

The common purslane (*P. oleracea* L) is a herbaceous succulent annual plant from the Portulacaceae family, native to the Middle East and India [1, 2]. It may be found on roadsides, gardens, and cultivated areas in the tropical and subtropical regions [3, 4]. There are various cultivars of *P. oleracea* distributed worldwide, mainly with morphological differences, with the common purslane having green-red stems, obovate leaves, yellow flowers, and single-layered petals, while the ornamental purslane produces flowers of different colors [1]. The stems and leaves have a slightly acid and salty taste and are usually consumed in salads, soups, and stews [5, 6]. It is an edible plant in regions of European, Mediterranean, African, and Asia countries and Australia [6]. In Brazil, *P. oleracea* is known as an “unconventional food plant”, a term referring to plants that are not part of the usual consumption of most of the population in a particular region, country, or even the planet because basic food is very homogeneous, with the use of few food species [7].

*P. oleracea* has a high nutritional value and many antioxidant properties due to its phenolic compound and omega-3 fatty acid abundance, particularly *α*-linolenic acid. It is well-known in traditional Chinese medicine [2]. for its use in diuretic, febrifuge, antiseptic, antispasmodic, and vermifuge treatments [8]. Among its various pharmacological properties are its anti-inflammatory [9], antioxidative [10], renoprotective [11], neuroprotective [12], hepatoprotective [13], and muscle-relaxing effects [14].

Anticarcinogenic activities have been reported for *P. oleracea*. Investigations were carried out to screen the activities for antihepatocellular carcinoma [15, 16], colon cancer [17], glioblastoma multiforme [18], ovarian cancer [19] sarcoma [20], lung cancer [16], anti-cervical [21], gastric cancer [22], and pancreatic cancer [23]. *P. oleracea* contains bioactive compounds with antioxidant properties, act on metastasis and invasion, modulate the immune system, and inhibit tumor formation [19, 24, 25, 4].

Thus, this mini-review aimed to assemble the anti-cancer effects of bioactive compounds of P. oleracea, demonstrating the molecular mechanisms and the potential for the development of functional food products with anticancer properties.

## 2. The Nutritional Value and Bioactive Compounds of *P. Oleracea*

Proximate analyses of *P. oleracea* components including leaves, seeds, stems, buds, and flowers, have been performed. Ash, fiber, protein, and fat approximate contents of P. oleracea leaves as 20.56%, 36.27%, 12.82%, and 3.75%, respectively, are found on a dry matter basis [26]. *P. oleracea* also contains minerals in its leaves with concentration values approximate such as potassium (3710 mg/100 g of dry matter), calcium (2390 mg/100 g), nitrogen (2170 mg/100 g), magnesium (580 mg/100 g), phosphorus (350 mg/100 g), sulfur (200 mg/100 g), iron (32.4 mg/100 g), manganese (5.8 mg/100 g), boron (2.8 mg/100 g), zinc (2 mg/100 g), and copper (1.1 mg/100 g) [26]. This study showed higher levels of potassium, calcium, magnesium, phosphorus, and iron when compared to those of spinach (336 mg/100 g of dry matter, 98 mg/100 g, 82 mg/100 g, 25 mg/100 g, and 0.4 mg/100 g, respectively) [27].

*P. oleracea* contains high amounts of Omega-3 fatty acids, as discussed by Siriamornpun and Suttajit [28] that found higher levels of Omega-3 fatty acids in fresh leaves, with 523.146 ± 2.29 mg/100 g, while, for stems and flowers, the authors reported 148.87 ± 3.30 mg/100 g and 216.17 ± 1.16 mg/100 g, respectively. Other plants (analysis of leaves in dry matter) contain lower levels of Omega-3 fatty acids than *P. oleracea*, such as mint (194.9 mg/100 g), watercress (179.6 mg/100 g), spinach (129.2 mg/100 g), parsley (124.8 mg/100 g), and broccoli (110.3 mg/100 g) (analysis of leaves in dry matter) [29]. Omega-3 fatty acids may have pharmacological effects such as anti-hyperlipidemic, antimicrobial, anti-inflamatory, neuroprotective and nephroprotective activities [3, 30, 31, 32, 11]. *P. oleracea* also contains high levels of tocopherols, vitamin A, *β*-carotene and ascorbic acid [3, 32–34]. Antimicrobial and antioxidant activities were related to these compounds [3, 33].

High concentrations of oxalic acid have also been detected in *P. oleracea.* The intake of oxalic acid provided by the diet with *P. oleracea* may form complexes with minerals such as calcium and iron (insoluble salts) or sodium, magnesium, and potassium (soluble salts), reducing their bioavailability and possibly leading to the development of kidney stones through the formation of calcium oxalate crystals [35]. Thus, consumption of *P. oleracea* should be moderated by individuals with a propensity to develop kidney stones. Amounts of 23.45 ± 0.45 g, 5.58 ± 0.18 g, and 9.09 ± 0.12 g of total oxalates per kilogram of fresh weight oxalates were obtained in fresh leaves, stems, and buds, respectively, with 75.0% being soluble oxalates in the stems and buds, and only 27.5% in the leaves [36]. The authors reported a 66.7% reduction (*p* < 0.001) of soluble oxalates after cooking the leaves for a short time, discarding the water, and pickling them with white vinegar [36]. Some other bioactive compounds from secondary metabolism of *P. oleracea* such as flavonoids, alkaloids, terpenoids and their pharmacological activity can be seen in Table 1.

Flavonoids (a class of phenolic compounds) in *P. oleracea* were associated with anti-fertility, antimicrobial, antioxidant and antidiabetic effects [37–40]. Combined effects of polyunsaturated fatty acids, flavonoids and polysaccharides on hypoglycaemic, hypolipidaemic and insulin resistance reducer effects through ingestion of P. oleracea seeds in clinical test with humans were observed [40]. Other phenolic compounds (Polyphenols and phenolic acids) in *P. oleracea* have antioxidant and antimutagenic effects [41–43].

Other bioactive compounds with pharmacological importance in *P. oleracea* are alkaloids and terpenes. Anticancer, anti-inflamatory and antioxidant effects were described for alkaloids found in this plant while hepatoprotective, antibacterial, antifungal and anti-hypoxia effects were described for terpenes of *P. oleracea* [44–48].

## 3. Potential Antioxidant of the *P. Oleracea*

This plant is rich in antioxidants such as vitamin A, tocopherols, ascorbic acid, beta-carotene, and phenolic compounds [33, 49]. Beta-carotene was found in *P. oleracea* with content ranging from 21 *μ*g/g to 30 *μ*g/g of fresh mass in leaves and 3.6 *μ*g/g to 6.5 *μ*g/g of fresh mass in stems [50]. The antioxidant potential was measured at different growth stages (15, 30, 45, and 60 days) of aerial parts of *P. oleracea* [49]. The total phenolic content (TPC) for the young shoots at 15 days was significantly lower than at 30, 45, and 60 days, while the ascorbic acid content (AAC) did not show a significant decrease from the developing to the mature stage. According to the study, the IC50 value of 1,1-diphenyl-2-picrylhydrazyl (DPPH) free radical scavenging activity ranged from 1.30 ± 0.04 mg/ml (60 days) to 1.71 ± 0.04 mg/ml (15 days), while the ascorbic acid equivalent antioxidant content (AEAC) values ranged from 229.5 ± 7.9 mg AA/100 g (15 days) to 319.3 ± 8.7 mg AA/100 g (60 days), the TPC varied from 174.5 ± 8.5 mg GAE/100 g (15 days) to 348.5 ± 7.9 mg GAE/100 g (60 days), the AAC varied from 60.5 ± 2.1 mg/100 g (60 days) to 86.5 ± 3.9 mg/100 g (15 days), and the ferric reducing antioxidant power (FRAP) ranged from 1.8 ± 0.1 mg GAE/g (15 days) to 4.3 ± 0.1 mg GAE/g (60 days). Thus, mature plants (60 days) of *P. oleracea* had higher TPC and antioxidant activities than immature plants.

The dry weights of the samples (leaves, flowers, and stems) from two different locations were investigated for potential antioxidant activity by Silva and Carvalho [41], who found that stems had a higher total phenolic content and total antioxidant activity than the flowers and leaves. The oil from seeds, leaves, and stems of *P. oleracea* were analyzed and found that the peroxide value was significantly higher for seed oil and the lowest for stem oil [51]. Furthermore, the highest ascorbic acid content was found for *P. oleracea* seed oil (41.67%), followed by leaf oil (32.29%), and the highest DPPH was obtained for leaf oil (12.55%), followed by seed oil (2.05%). Values for lettuce (IC50 = 17.07 mg/ml), artichoke (IC50 = 18.14 mg/ml), turmeric (IC50 = 21.14 mg/ml), spinach (IC50 = 22.87 mg/ml), and escarole (IC50 = 32.2 mg/ml) were reported by Tiveron et al. [52], showing that *P. oleracea* presents the lowest IC50 necessary to reduce 50% of DPPH free radicals.

## 4. Functional Food Products and *P. Oleracea*

The *P. oleracea* plant may be used as an ingredient in functional food products due to its nutritional value and bioactive compounds that will be incorporated into the formulations.

The use of the *P. oleracea* plant as food may not only enhance the nutrients and bioactive composition of functional products but also influence their sensory and technological characteristics. Although it is well-known that sensory acceptance by consumers is essential for a product's commercial success on the market, few studies in the literature have reported the application of *P. oleracea* in products and its performance or the sensory profile of such products.

Regarding the technological aspect, the incorporation of the durum wheat flour with 5% of *P. oleracea* to bread resulted in the improvement of the rheological characteristics, an increase in antioxidant properties, and a decrease in the Omega-6-to-Omega-3 ratio, which is beneficial for human health, in addition to improving the sensorial quality [53].

The durum wheat spaghetti fortified with 10% of *P. oleracea,* a potential functional food, was appreciated by consumers. It showed a high concentration of *α*-linolenic acids (Omega-3), total phenolic compounds, and antioxidant properties, so that, considering 100 g of pasta per day, it is possible to obtain 75 mg of essential linoleic acid and 9 mg of linolenic acid, along with a four-fold increase in total phenolic compounds [54]. The Omega-3 fatty acids can also inhibit carcinogenesis and slow tumor growth, as demonstrated by *in vitro*, *in vivo*, and clinical investigations [55].

The analysis of bread incorporated with four different concentrations of *P. oleracea* powder (0%, 5%, 10%, and 15%) showed increasing water absorption capacity, stability under the mixer, and softening levels as the *P. oleracea* powder concentration in the samples increased. The protein, fat, total ash, moisture, and fiber contents also increased along with the *P. oleracea* concentrations [56]. However, the bread with 15% of *P. oleracea* powder showed a decreased farinograph quality number and presented the lowest scores for sensory properties and color, taste, texture, and overall liking. The optimized formulation containing 10% of *P. oleracea* powder had the highest acceptance.

*P. oleracea* has also been used to produce powder mixtures with two other plant species, *Amaranthus hybridus* L. and *Chenopodium berlandieri* L. The powder mixtures containing *P. oleracea* showed more significant contents of phenolic compounds, with an increase in the antioxidant activity [57].

Another innovative functional product assessed was a fermented *P. oleracea* juice added with a selected lactic acid bacteria. Results demonstrated an increase in total antioxidants, preserved vitamin C, A, and E levels, and increased contents of vitamin B2 and phenolic compounds. In addition, decreased levels of pro-inflammatory mediators and reactive oxygen species were observed, with a consequent increase in the restorative characteristics of the use of *P. oleracea* juice for intestinal inflammation and epithelial injury [58].

The combination of yogurt or coconut plant extract or coconut cream with fresh leaves of *P. oleracea* reduced the overall oxalate content by simple dilution. The soluble oxalate content decreased from 53.0% to 10.7% when *P. oleracea* leaves were added to yogurt. However, the coconut plant extract and coconut cream had no effect on the percentage of soluble oxalate content but provided the mixture with an acceptable flavor [59].

The addition of fresh purslane leaves (ranging from 1% to 10%, w/w) to tomato sauces resulted in a decrease of total soluble solids from 9.57°Bx to 9.20°Bx, beneficially impacting sugar reduction. On the other hand, the amount of protein significantly increased from 0.12% to 1.83% from the lowest to the highest concentrations, respectively [60].

## 5. Bioactive Compounds of *P. Oleracea* on Anticancer Activity

*P. oleracea* presents phytochemicals and nutrients associated with anticarcinogenic properties. The 12% reduction in the activity of the mutagenic nitrosation mixture may be attributed to the ascorbic acid (vitamin C), *α* and *β*-carotene, chlorophyll, and polyphenols of the *P. oleracea* extract obtained through a standard juice extractor [42].

Phenolic compounds such as kaempferol and apigenin from a hydroethanolic extract of *P. oleracea* have effects *in vitro* against human glioma cells, and homoisoflavonoids showed *in vitro* selective cytotoxic activity for SF-268, NCI-H460, and SGC-7901 cell lines, as shown Table 2 [18, 61].

Polysaccharides from *P. oleracea* act on free radicals through the antioxidant mechanism, modulating the immune system, which may be preventive and therapeutic in rat ovarian and gastric cancer and mouse cervical cancer and sarcomas, as shown Table 2 [19–22, 62].

Another bioactivity from *P. oleracea* is portulacacerebrosie A, a cerebroside compound that suppresses the invasion and metastasis of liver cancer HCCLM3 cells and acts in leucocythemia treatment are show in Table 2 [24, 63].

Polysaccharides showed activity against ovarian cancer by inhibiting the red blood cell (RBC) hemolysis in the spleen, thymocyte, and T and B lymphocyte proliferation [19]. These compounds also act against cervical cancer through Sub-G1 phase cell cycle arrest triggering DNA damage, inhibit the growth of transplantable sarcoma 180, increase the number of white blood cells (WBC), CD4+ T-, the CD4^+^/CD8^+^ ratio, IL-12, and TLR-4, decrease IL-10 and HeLa cell proliferation, reduce the production of cytokine/chemokine and the expression levels of CD80, CD86, CD83, Bax, and downregulate the Bcl-2 level in a concentration-dependent manner. In addition, polysaccharides inhibit the protein expression levels of TLR4, myeloid differentiation primary response 88 (MyD88), TNF receptor associated Factor 6 (TRAF6), activator protein-1 (AP-1), and factor nuclear kappa B (NF-*κ*B) subunit P65 [20, 21, 63, 64].

In gastric cancer, interleukins (IL-2 and IL-4) and TNF-*α* were enhanced by polysaccharides that also provide dose-dependent protection against N-methyl-N'-nitro-N-nitrosoguanidine (MNNG) induced oxidative injury by enhancing Superoxide dismutase (SOD), catalase (CAT), and glutathione (GSH-Px) [22]. In addition to acting against ovarian, gastric, and cervical cancer, polysaccharides also work against intestinal cancer by stimulating the TLR4-PI3K/AKT-NF-*κ*B signaling pathway and Anti-NF-*κ*B activity along with two upstream ROS and NO mechanisms [18, 62], showing the importance of studying these molecules in *P. oleracea* matrices.

The cerebroside compound, Portulacerebroside A, affects leukemia and cervical, liver, esophageal, breast, and colon cancer and cancer stem cells [16, 24, 63, 65, 66]. Some mechanisms involved with Portulacerebroside A have increased RNA expressions and protein levels of Bax/Bcl-2, caspase-3, and caspase-9, protein expression levels of TIMP-2 and nm23-H1, inhibition of the mRNA expression of MTA1, MMP-2, and MMP-9, RhoA, Rac1/Cdc42, MMP-2, and downregulation of the expression of the Notch1 and *β*-catenin genes.

Alkaloids inhibited lung and breast cancer through moderate cytotoxic activities against A549, weak cytotoxic activities against K562, and low cytotoxic activity against MCF-7 and MDA-MB-435 cells.

**Figure 1:**
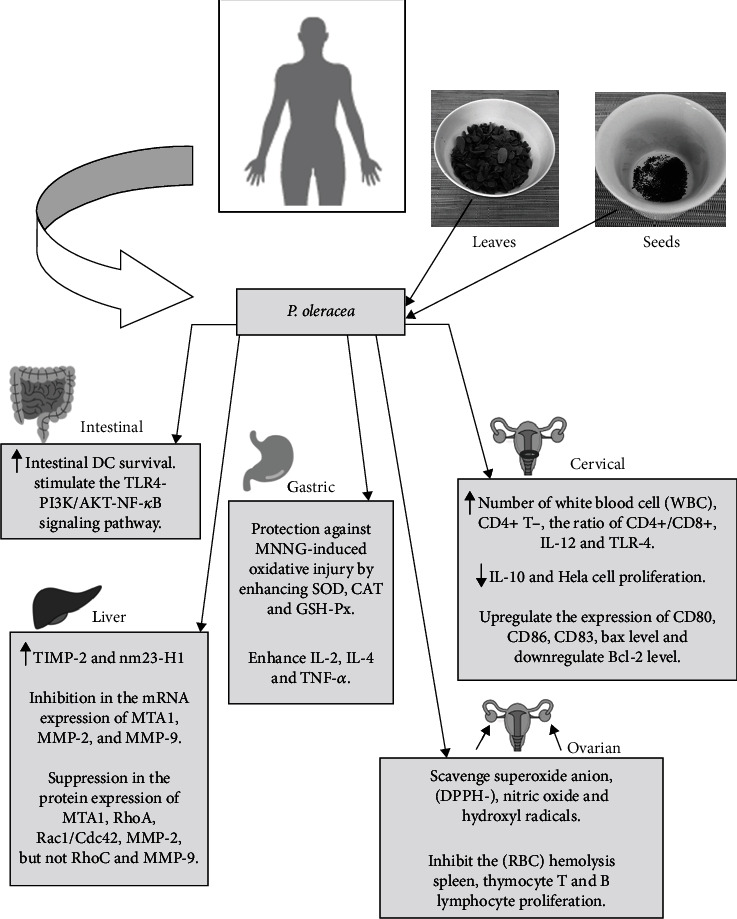
Some possible mechanisms of *P. oleracea* for anticancer activity.

Some possible mechanisms of *P. oleracea* for anticancer activity are represented in Figure 1. The bioactivity of *P. oleracea* and the potential to develop new products from this underused plant in some regions deserve attention regarding its valorization as a functional food and its pharmacological properties. Different anticancer mechanisms of *P. oleracea* were explored and reported in this review. Aqueous extracts, seed oil, and hydroethanolic extracts present cytotoxicity to cancer cell lines while chloroform extract does not have cytotoxic activity [67]. Further studies will be needed to determine anticancer activity in particular food matrices and beverages.

## 6. Conclusion

The *P. oleracea* plant may be promising for developing and innovating potential functional food products. The high levels of antioxidants such as phenolic compounds, carotenoids, and other nutrients such as minerals and Omega-3 fatty acids are supported by functional food studies. Research has indicated the anticancer activity of *P. oleracea* extracts. Polysaccharides, some phenolic compounds, alkaloids, and cerebrosides detected in *P. oleracea* and contained in aqueous extracts, seed oil, and hydroethanolic extracts are associated with inhibition and prevention of carcinogenesis. However, more studies are needed to prove the anticancer activity of food products containing *P. oleracea* as an ingredient to promote health benefits to the consumers.

## Figures and Tables

**Table 1:** Some classes of bioactive compounds from secondary metabolism of P. oleracea and their pharmacological activity.

Compounds	Plant structure	Form (fresh or dry)	Pharmacological activity	References
Flavonoids	Aerial part	Dry	Antifertility	[37]
	Aerial part	Dry	Antimicrobial	[38]
	Leaves	Fresh	Antioxidant	[39]
	Seeds	Dry	Antidiabetic	[40]

Polyphenols	Leaf, steam and flower	Dry	Antioxidant	[41]
	Whole plant	Fresh	Antimutagenic	[42]

Phenolic acids	Aerial parts	Dry	Antioxidant	[43]
Alkaloids	Aerial part	Dry	Anticancer	[44]
	Whole plant	Fresh	Anti-inflamatory	[45]
	Whole plant	Dry	Antioxidant	[46]

Terpenes	Whole plant	Dry	Hepatoprotective, antibacterial and antifungal	[47]
	Aerial part	Dry	Anti-hypoxia	[48]

**Table 2:** Bioactive compounds of *P. oleracea*, types of extracts, and molecular mechanisms for cancer inhibition.

Experimental model		Compounds	Types of extract	Types of cancer inhibited	Mechanisms and results	References
*In vitro*	*In vivo*					
	Rats	Polysaccharides	Aqueous extract	Ovarian	Scavenge superoxide anion, (DPPH-), nitric oxide, and hydroxyl radicals Inhibit RBC hemolysis Spleen, thymocyte, T and B lymphocyte proliferation	[19]
Human cancer cell lines SF-268, NCI-H460, K-562, SGC-7901, and SMMC-7721		Homoisoflavonoids	Hydroalcoholic extract		Homoisoflavonoids showed in vitro cytotoxic activities towards four human cancer cell lines	[61]
Treatment of HeLa cell	Mice	Polysaccharides	Aqueous extract	Cervical	Sub-G1 phase cell cycle arrest, triggering DNA damage Inducing apoptosis	[21]
	Mice	Polysaccharides	Aqueous extract		Inhibit the growth of transplantable sarcoma 180 Increase in the number of white blood cells (WBC) and CD4+ T-lymphocytes Increase in the CD4^+^/CD8^+^ ratio	[20]
	Rats	Polysaccharides	Aqueous extract	Gastric	Interleukin-2 (IL-2), interleukin-4 (IL-4), and tumor necrosis factor-alpha (TNF-*α*) was enhanced Provide dose-dependent protection against MNNG-induced oxidative injury by enhancing SOD, CAT, GSH-Px	[22]

Human lung (K562 and A549) and breast (MCF-7 and MDA-MB-435) cancer cell lines		Alkaloids	Hydroalcoholic extract	Lung Breast	Moderate cytotoxic activities against A549 and weak cytotoxic activities against K562. The compounds showed low cytotoxic activity against MCF-7 and MDA-MB-435 cells.	[54]
Human hepatocellular carcinoma cells			Seed alcoholic extract	Hepatocellular	Significantly reduced the cell viability of HepG2.	[15]
The uterine cervical carcinoma (U14) cell line		Polysaccharides	Aqueous extract	Cervical	Upregulated the expression of CD80, CD86, CD83 Increase in IL-12, TLR-4, Decrease in IL-10	[29]
Human HL60 cell line		Portulacerebroside A	Aqueous extract	Leukemia	Mitochondrial membrane potential ROS accumulated Increase in RNA expressions and protein levels of Bax/Bcl-2, caspase-3, and caspase-9 ERK1/2, JNK1/2 and p38 MAPK pathway were blocked	[49]
HepG2 and A-549 cell lines			Seed oil	Liver Lung	Significant cytotoxicity and inhibition of growth of the liver cancer (HepG2) and lung cancer (A-549) cell lines	[20]
Human liver cancer HCCLM3 cells		Portulacerebroside A	Aqueous extract	Liver	Increase in RNA and protein expression levels of TIMP-2 and nm23-H1 Inhibition of the mRNA expression of MTA1, MMP-2, and MMP-9 Suppression of the protein expression of MTA1, RhoA, Rac1/Cdc42, MMP-2, but not RhoC and MMP-9	[23]
Cervical cancer HeLa cells, esophageal cancer Eca-109 cells and breast cancer MCF-7 cells			Seed oil	Cervical Esophageal Breast	Stronger inhibitory effect on the proliferation of MCF-7 cells and significantly inhibited the proliferation of HeLa cells and Eca-109 cells	[51]
PANC-1 cancer cell line			Aqueous extract	Pancreatic	Significant effect on apoptosis in pancreatic cell line and high expression of P53 and reduction of CDK gene expression	[23]
Human colon adenocarcinoma (HCT-15) and normal (Vero) cell line			Chloroform extract	Colon adenocarcinoma	Chloroform extract does not have cytotoxic activity and was not safe to normal Vero cell line.	[67]
Colon cancer cells (HT-29) and HT-29 cancer stem cells			Ethyl alcohol extract	Colon Stem cells	Inhibited the proliferation of both HT-29 cancer cells and HT-29 cancer stem cells Significantly decreased the expression of the Notch1 and *β*-catenin genes in both cell types	[52]
The human cervical cancer HeLa cells.		Polysaccharides	Aqueous extract	Cervical	Decrease HeLa cell proliferation Upregulate Bax level and downregulate Bcl-2 level in a concentration-dependent manner Inhibit the protein expression levels of TLR4, MyD88, TRAF6, AP-1 and NF-*κ*B subunit P65 Reduce the production of cytokine/chemokine	[50]
The mouse cervical carcinoma U14 cells		Polysaccharides	Aqueous extract	Intestinal	Dendritic cell (DC) apoptosis in U14-bearing mice Increase intestinal DC survival Stimulate the TLR4-PI3K/AKT-NF-*κ*B signaling pathway	[48]
Human glioblastoma cancer cell line (U-87)			Hydroethanolic extract		Cytotoxicity and apoptogenic effects Anti-NF-*κ*B activity along with two upstream ROS and NO mechanisms	[17]

## Data Availability

The data used to support the findings of this study are included within the article.
